# Toward a pluri-component, multimodal, and dynamic organization of the ventral semantic stream in humans: lessons from stimulation mapping in awake patients

**DOI:** 10.3389/fnsys.2013.00044

**Published:** 2013-08-26

**Authors:** Hugues Duffau, Guillaume Herbet, Sylvie Moritz-Gasser

**Affiliations:** ^1^Department of Neurosurgery, Hôpital Gui de Chauliac, CHU MontpellierMontpellier, France; ^2^Team “Plasticity of Central Nervous System, Stem Cells and Glial Tumors,” INSERM U1051, Institute for Neuroscience of Montpellier, Hôpital Saint EloiMontpellier, France; ^3^Department of Neurology, Hôpital Gui de Chauliac, CHU MontpellierMontpellier, France

**Keywords:** ventral stream, semantic processing, anatomo-functional connectivity, awake surgery, brain electrostimulation mapping, subcortical pathway

For many decades, neural basis underlying cognitive functions was conceived in a localizationist framework. Owing to the development of connectomics, an alternative hodotopical account was proposed, in which brain functions are sub-served by the interactions of large-scale distributed and parallel subnetworks (Catani, 2007; de Benedictis and Duffau, 2011). In this setting, the processing of visual information is divided in a dorsal stream dedicated to the analysis of the spatial position (“where”) and in a ventral stream specialized in object identification (“what”; Underleider and Haxby, 1994). By analogy, a dual-stream model for auditory language processing was suggested, with a dorsal stream involved in mapping sound to articulation and a ventral stream involved in mapping sound to meaning (Hickok and Poeppel, 2004). Nonetheless, the neural structures supporting the ventral route is still controversial. We have recently proposed a new model of language, in which the subcortical anatomical constraints have been incorporated (Duffau et al., 2013): beyond a well-recognized dorsal phonological/articulatory stream underlain by the superior longitudinal fascicle, the neuroanatomy subserving a parallel ventral stream involved in multimodal semantics was described.

Here, our purpose is to detail the dynamic functional anatomy of this multi-component ventral route, constituted by direct and indirect pathways (explaining a possible compensation following brain injury) and implied in pluri-modal semantic processes—i.e., in verbal and non-verbal comprehension, control and noetic consciousness.

## Structural considerations: the multi-component tracts subserving the ventral stream

Anatomically, the ventral route connects the occipital and posterior temporal areas with the frontal lobe. This ventral stream is referred by some authors as “extreme capsule” with reference to connectivity studies in primate (Makris and Pandya, 2009). In our view, it is more adapted to speak about fascicles rather than “extreme capsule,” because the latter only considers a discrete anatomical structure while the former considers actual neural pathways with their cortical termination, in a hodotopical view. Indeed, if one takes account of the sole subcortical region without any considerations regarding the cortical epicenters connected by the white matter tracts, it does not allow the understanding of the whole eloquent network.

In this mind, using both anatomic dissection and tractography, we demonstrated that the ventral stream was under-lain by direct and indirect pathways. The direct pathway is represented by the inferior fronto-occipital fascicle (IFOF). This IFOF was never described in animals, explaining the debate about its role. In humans, the IFOF is a ventral associative bundle that connects the occipital lobe, parietal lobe, and the postero-temporal cortex with the frontal lobe. Recent anatomic studies using post-mortem white-matter dissection (Martino et al., 2010) and DTI works have investigated the main course of the IFOF (Sarubbo et al., 2013). From the posterior cortex, it runs within the sagittal stratum in the superior and lateral part of the atrium; it reaches the roof of the sphenoidal horn in the temporal lobe; it joins the ventral part of the external/extreme capsule and it runs under the insula at the posterior two-thirds of the temporal stem; then it joins the frontal lobe (Martino et al., 2010). Two layers of the IFOF have been described (Sarubbo et al., 2013). The superficial and dorsal layer connects the posterior portion of the superior and middle occipital gyri, the superior parietal lobule, and the posterior part of the superior temporal gyrus to the inferior frontal gyrus (pars triangularis and opercularis). The deep and ventral subcomponent connects the posterior portion of the inferior occipital gyrus, the posterior temporal-basal area including the Fusa (fusiform area at the occipito-temporal junction), and the posterior part of the middle temporal gyrus to the frontal lobe—orbito-frontal cortex, middle frontal gyrus, and dorsolateral prefrontal cortex.

In parallel, the ventral stream is subserved by an indirect pathway, constituted by the anterior part of the inferior longitudinal fascicle (ILF; running below the IFOF), that links the posterior occipito-temporal region (Fusa), and the temporal pole (TP), then relayed by the uncinate fasciculus (UF), that connects the TP to the basifrontal areas by running within the anterior third of the temporal stem (in front of the IFOF; Mandonnet et al., 2007). Of note, the posterior part of the ILF links the occipital lobe to the posterior occipito-temporal junction (visual object form area; Mandonnet et al., 2009). This means that this indirect route connects the occipital/Fusa to the orbito-frontal cortex—thus partially overlapped with the IFOF. Finally, another pathway has recently been described in humans: the middle longitudinal fascicle (MdLF). It connects the angular gyrus with the superior temporal gyrus up to the TP and courses under the superior temporal sulcus, lateral and superior to the IFOF (Menjot de Champfleur et al., 2013).

**Figure 1:**
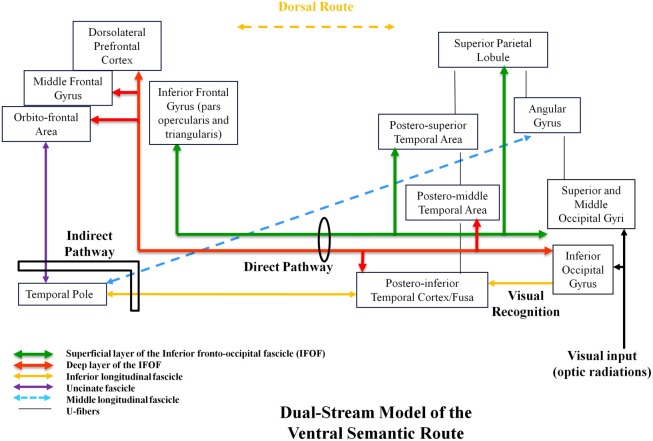
**Proposal of a dual-stream model of the ventral amodal semantic route, with incorporation of anatomic constraints, elaborated on the basis of structural–functional correlations provided by intraoperative DES**.

In summary, the ventral stream has a complex architecture based on several bundles allowing multiple connections between the posterior parieto-occipito-temporal cortex and the anterior brain (TP and frontal lobe). This multi-component anatomy is crucial to better understand the functional role of this large ventral sub-network (Figure 1).

## Functional considerations: the ventral stream is involved in multimodal semantic processing

### The method of intrasurgical stimulation mapping

Direct electrical stimulation (DES) in patients undergoing brain surgery offers a unique opportunity to investigate functional anatomy. It has become common clinical practice to awaken patients in order to assess the functional role of restricted cortical and subcortical regions, and to avoid neurological impairments. Patients perform cognitive tasks while DES temporarily inactivates discrete brain areas: if the patients produce wrong response, the stimulated site is preserved. DES interacts locally with a small cortical or axonal site, but also non-locally, as the focal perturbation disrupts the whole (sub)network sustaining a given function. Therefore, conversely to functional neuroimaging, DES induces a transient virtual lesion, by inhibiting a sub-circuit during a few seconds. By gathering all cortical and axonal sites where the same type of errors were observed when stimulated, one can build up the sub-network of the disrupted sub-function. DES identifies with a great accuracy (about 5 mm) and reproducibility, *in vivo* in humans, the structures—cortex and white matter tracts—crucial for cognitive functions (Duffau, 2011). Combining transient disturbances elicited by DES with the anatomical data provided by pre-and post-operative MRI enables to perform reliable anatomo-functional correlations. We used DES to study the function of the ventral stream.

### The results of structural-functional correlations: the crucial role of IFOF in semantics

During picture naming, DES of the IFOF, at least in the left dominant hemisphere, elicited semantic paraphasias either associative (e.g., /key/ for /padlock/) or coordinate (e.g., /tiger/ for /lion/) in more than 85% of cases—whatever the portion of the IFOF stimulated (parieto-occipital junction, temporal, subinsular, or frontal part) (Duffau et al., 2005). These language disorders were mainly generated by stimulating the superficial layer of the IFOF. Of note, semantic paraphasias were never observed during stimulation of the dorsal route (superior longitudinal fascicle; Maldonado et al., 2011).

Furthermore, DES of the IFOF induced non-verbal comprehension disturbances in more than 90% of cases during non-verbal semantic association test—e.g., Pyramid and Palm Trees Test (Howard and Patterson, 1992). The patients were not able anymore to make a semantic choice during DES, with some of them still able to join a short verbal description of their feelings, like “I don't know at all,” “what do I have to do?,” “I don't understand anything” (Moritz-Gasser et al., 2013). These comprehension disorders were mainly generated by stimulating the deep layer of the IFOF, thus with a double dissociation: semantic paraphasia with normal non-verbal semantic choice during DES of superficial IFOF and *vice versa* during DES of deep IFOF. Thus, we suggest the existence of a superficial component involved in verbal semantics and a deep component involved in amodal semantic processing.

These data are in agreement with the cortical terminations of the IFOF (prefrontal, temporal-basal, and parietal areas), that correspond with the cortical network involved in semantic control (Whitney et al., 2011). Consequently, we have recently proposed an original anatomo-functional model of semantic processing, in which the crucial pathway is represented by the IFOF. In this model, visual information is processed at the level of the occipital and temporal-basal associative cortices, and auditory information is processed at the level of the temporal and parietal associative cortices. They are transmitted directly on an amodal shape to the prefrontal areas which exert a top down control over this amodal information in order to achieve a successful semantic processing in a given context. DES of this fascicle generates a disruption of these rapid direct connections. The transient semantic disorganization observed when stimulating the IFOF would therefore be caused by a dis-synchronization within this large-scale network, interrupting simultaneously the bottom up transmission and the top down control mechanisms (Moritz-Gasser et al., 2013). This fits well with the fact that stimulation of the IFOF can also induce verbal perseveration, which could be due to impairment of an inhibitory system that causes an increase in facilitatory activity and involuntary recall of recently established memory—supporting the idea that IFOF could be involved in semantic control (unpublished data). Interestingly, recent neuroimaging study has shown that the IFOF was implied in semantic memory (De Zubicaray et al., 2011).

To sum up, in our opinion, IFOF might play a crucial role not only in verbal and non-verbal semantic processing, but also in the awareness of amodal semantic knowledge, namely noetic consciousness. From a phylogenetic perspective, because recent studies in the primate failed to identify this tract, we suggest that the IFOF is the proper human fascicle. This multi-function fascicle allows human to produce and understand language, to manipulate concepts, to apprehend and understand the world (i.e., metalinguistics, conceptualization, and awareness of knowledge) and it contributes to make the human what he is, with his infinite wealth of mind.

### A multi-bundles dynamic organization opening the window to network reshaping

The functional role of the indirect pathway is still matter of debate. On one hand, this indirect route connects areas involved in semantic processing such as Fusa and lateral frontal cortex (Vigneau et al., 2006). Moreover, the major cortical relay between the ILF and UF is the TP, which is a “hub,” i.e., a functional epicenter allowing a plurimodal integration of the multiple data coming from the unimodal systems (subserved by ILF, UF, and MdLF)—explaining its role in semantics and its implication in semantic dementia when (bilaterally) damaged (Holland and Lambon-Ralph, 2010). On the other hand, except for the posterior part of the ILF for which injury generates alexia (Mandonnet et al., 2009), the indirect pathway can be functionally compensated when (unilaterally) damaged. Indeed, DES of both the anterior ILF and UF never elicited any naming or non-verbal semantic disorders in our experience (Mandonnet et al., 2007; Duffau et al., 2009). This was also confirmed by functional recovery following anterior temporal lobectomy in tumor and in epilepsy surgery (Duffau et al., 2008). Even if very mild and selective deficit may persist, as concerning proper name retrieval after resection of the UF (Papagno et al., 2011), this is a good illustration of the concept of “subcortical plasticity,” in which a sub-network (IFOF, direct pathway) is able to bypass another sub-network (indirect pathway) and to functionally compensate it (Duffau, 2009). It is nonetheless possible that this indirect pathway is involved in other functions, as emotion processing and behavior. Similarly, DES of MdLF and resection of its anterior part failed to induce any functional disorders (De Witt Hamer et al., 2011), demonstrating that this fascicle converging to the TP can also be compensated.

## Conclusion

We propose a dynamic (sub)dual-route model underlying the ventral stream itself, constituted by a direct and essential pathway (multi-component IFOF) and an indirect and compensable pathway (ILF/TP/UC ± MdLF; Figure 1). Based on unique data issued from intraoperative DES in awake patients, we assume that this ventral route has a plurimodal semantic role (verbal and non-verbal processing) and an amodal role (control and consciousness). Further studies are needed to validate this model and to bring new insights into a likely bilateral distribution of the ventral stream—and into its possible involvement in spatial cognition and mentalizing.

## Funding

Guillaume Herbet is supported by the Association pour la Recherche sur le Cancer (aides individuelles n = ° DOC20120605069).
